# Overview of β-Glucans from *Laminaria* spp.: Immunomodulation Properties and Applications on Biologic Models

**DOI:** 10.3390/ijms18091629

**Published:** 2017-09-06

**Authors:** Patrícia de Souza Bonfim-Mendonça, Isis Regina Grenier Capoci, Flávia Kelly Tobaldini-Valerio, Melyssa Negri, Terezinha Inez Estivalet Svidzinski

**Affiliations:** 1 Graduate Program in Health Sciences, Department of Clinical Analysis and Biomedicine, State University of Maringa, Paraná 87020-900, Brazil; psbmendonca@gmail.com; 2 Graduate Program in Biosciences and Pathophysiology, Department of Clinical Analysis and Biomedicine, State University of Maringa, Paraná 87020-900, Brazil; isiscapoci@gmail.com (I.R.G.C.); ftobaldini@gmail.com (F.K.T.-V.); 3 Department of Clinical Analysis and Biomedicine, State University of Maringa, Paraná 87020-900, Brazil; melyssanegri@gmail.com

**Keywords:** *Laminaria*, β-glucan, glucans, immunomodulatory properties, defense

## Abstract

Glucans are a group of glucose polymers that are found in bacteria, algae, fungi, and plants. While their properties are well known, their biochemical and solubility characteristics vary considerably, and glucans obtained from different sources can have different applications. Research has described the bioactivity of β-glucans extracted from the algae of the *Laminaria* genus, including in vivo and in vitro studies assessing pro- and anti-inflammatory cytokines, vaccine production, inhibition of cell proliferation, and anti- and pro-oxidant activity. Thus, the objective of this article was to review the potential application of β-glucans from *Laminaria* spp. in terms of their immunomodulatory properties, microorganism host interaction, anti-cancer activity and vaccine development.

## 1. Introduction

Glucans are a heterogeneous group of glucose polymers that are found in bacteria, algae, fungi and plants. These polymers are involved in the cell wall structure and other biological functions. They are classified according to their interchain linkage as either α- or β-glucans. The common structure consists of a main chain comprising a β-(1,3) and/or β-(1,2); β-(1,4); and β-(1,6)-d-glucopyranosyl unit in a non-repeating but non-random order, together with side chains of various lengths. In addition, the structures can be linear, side-chain-branched, branch-on-branch and cyclic. [1,2]. There is considerable variation in the biochemical and solubility characteristics of β-glucans obtained from different sources [3]. High molecular weight glucans such as β-(1,3;1,6)-d-glucans (branch-on-branch) are present in fungi such as the *Saccharomyces cerevisiae* and are insoluble in water and alkali. On the other hand, glucans extracted from algae have a low molecular weight with frequent binding of the glucopyranosyl residues by β-1,3-d-glycosidic bonds or β-1,6-d-branched β-1,3-d-glycosidic bonds, and exhibit frequent solubility in water [4].

The biological actions of the different β-glucans represent a broad spectrum of health benefits, and their activity varies according to the molecular structure and solubility of each polymer [5,6,7]. In general, the benefits are linked to the stimulation of the immune system, notably through the increased regulation of the respiratory burst activity of macrophages [8]. Their action also extends to tumor suppression and antigenic stimulus for vaccines [9].

In recent years, the extraction of β-glucans from the seaweed of the *Laminaria* genus (denominated Laminarin or Laminaran (LAM)) has emerged. These glucans appear to have advantages in relation to glucans of other algae, because they have low molecular complexity, conferring benefits to biological activity, as bio functional food, ability to induce antibodies and have potent immunostimulatory effects, affecting both natural and adaptive immunity [2,4,5,10,11,12]. After carbon, Laminarin is the second largest storage component in brown algae [13]. Both in vivo and in vitro research on the bioactivity of this group of glucans has been carried out, and has included evaluations of pro- and anti-inflammatory cytokines [14,15,16,17], antioxidant activity [6,14], the inhibition of cell proliferation [18,19], vaccine production [20,21,22], and microorganism host interaction [5,6]. Due to these characteristics of Laminarin, the present review provides an overview of the potential applications of β-glucans extracted from the *Laminaria* spp. genus, highlighting their immunomodulatory properties and some applications in biologic models.

## 2. Molecular and Biochemical Characteristics of Laminarin (LAM)

**Figure 1:**
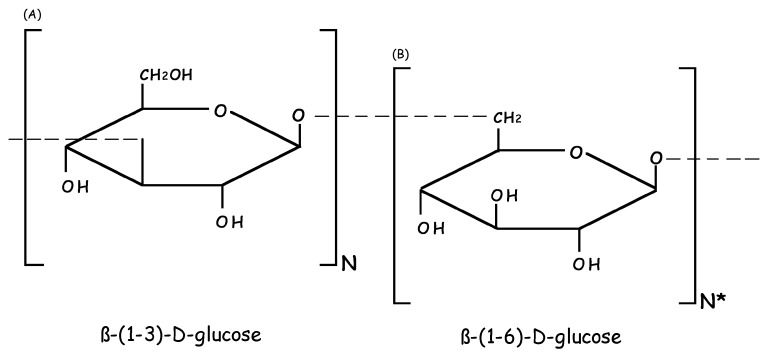
Schematic representation of the basic molecular structures of LAM: (**A**) β-(1-3)-d-glucose linked; and (**B**) β-(1-6)-d-glucose branch. N: number of specific repetitions of β-(1-3)-d-glucose linked; N *: number of specific repetitions of β-(1-6)-d-glucose branch, both according to the species *Laminaria* spp.

The molecular structure of the β-glucan known as LAM varies according to the species of *Laminaria* spp. from which the compound is extracted, and not all have been fully described in the literature. LAM has an average molecular weight of 5000 Da (ranging from 3400 to 7700 Da), and is known to be mainly constituted of a polysaccharide consisting of 25–50 glucose units that are linked by β-1,3-glycosidic bonds, often with β-1,6-branching (Figure 1). LAM can exhibit two types of chains, which differ in the reducing ends, with a glucose residue in the G-type LAM and mannitol residue in the M-type LAM [1,2,23]. The solubility of different molecules is influenced by the degree of branching of each. LAM from several species of *Laminaria* are water-insoluble and contain only linear β-1,3-linked residues, while water-soluble LAM contain significant levels of β-1,6-linked branches [1,2]. The constitution of the polysaccharides varies according to species, geographic location and season. LAM and fucoidan (FUC) are the main polysaccharides studied that have biological effects [24,25,26,27,28,29]. Three species of *Laminaria*, *L. digitata*, *L. hyperborea* and *L. japonica*,have been highlighted in studies as the source of these compounds.

*L. digitata* is common in the intertidal zone of rocky coastlines in the temperate northern hemisphere [30]. It is ecologically important as it provides a habitat for many smaller macroalgae [31] and a habitat and food for many marine animals (e.g.,fish, crustaceans, mollusks, and polychaetes) [32]. It plays an important role in iodine emissions and is a mediator of coastal iodine fluctuations [33]. LAM derived from *L. digitata* is water-soluble and contains a small number of β-(1-3)-linked side chains, and small but significant levels of β-(1-6)-linked branches [1].In contrast, LAM derived from *L. hyperborea* is water-insoluble and contains only linear β-(1-3)-linked residues.This LAM is found on the coast of Norway, Scotland and Ireland, and is commercially exploited by the hydrocolloid industry for alginate production [34].

The brown seaweed *L. japonica* is often used in food preparation in oriental countries as a health protector. It has been used as a popular therapeutic agent for phlegm elimination, detumescence and weight loss in traditional Chinese medicine for over a thousand years [35,36]. This LAM has also been investigated for the effects of its products such as crude soluble polysaccharides (SPSs), and ethanolic extract (EE) fractions of frond (kombu) and holdfast (ganiashi) [37].

## 3. Immunomodulatory Properties of LAM

The use of immunomodulators to enhance host defense responses is considered the most promising of various therapeutic options. Recent research has shown that LAM from *Laminaria* spp. has significant immunostimulatory activity both in vivo and in vitro [13,14,38]. We therefore selected publications found in different sources studied that used LAM in both in vitro (animal cells) and in vivo (animal) study models, as well as those that studied LAM in microorganism host interaction, highlighting its main immunomodulatory properties, as shown in Table 1.

### 3.1. Immunomodulation by LAM in Animal Cells

Some in vitro models have been employed to evaluate the potential immunostimulatory effect of LAM. In this context, professional phagocytes such as macrophages, monocytes, leukocytes and neutrophils are the main targets of the study. These cells were successfully implemented in an attempt to reveal the signaling pathways of these effects.

#### 3.1.1. Macrophages

Phagocytosis of microorganisms is the first and key feature of macrophage function in host defense and tissue homeostasis in different hosts [39,40]. These phagocytes are capable of producing chemical intermediates that can eliminate important pathogens. Lee et al. [38], working with RAW 264.7 mouse macrophages and different LAM concentrations (from 100 to 500 µg/mL from *L. digitata*), found that the immune stimulatory effects occurred through inflammatory mediators such as calcium, hydrogen peroxide (H_2_O_2_), nitric oxide (NO), cytokines (monocyte chemotactic protein-1 (MCP-1)), vascular endothelial growth factor (VEGF), leukemia inhibitory factor, granulocyte-colony stimulating factor (G-CSF), IL-6, macrophage inflammatory proteins (MIP-1α), and transcription factors (signal transducer and activator of transcription 1 (STAT1), STAT3, Jun, Fos, COX-2, and TLR2). The increase in NO concentration was attributed to calcium signaling, as when this pathway was inhibited, a decrease in NO production was observed. In addition, literature indicates that an increase in cytosolic calcium activates calcium-dependent transcription factors, including STAT1, STAT3 and activator protein 1 (Fos and Jun composite heterodimeric protein), subsequently increasing the transcription of pro-inflammatory target genes. [41], which corroborates the results found for the transcription pro-inflammatory target genes of STAT3, c-jun, c-fos and cyclooxygenase-2 (COX-2) from that study. The authors suggested that the activation of macrophages by LAM was not cytotoxic and therefore safer than the traditional activation by bacterial Lipopolysaccharide (LPS), a molecule present on the outer surface of gram-negative bacteria that can induce symptoms of acute bacterial infection and immune stress through the synthesis and release of inflammatory cytokines by stimulated macrophages [42,43].

Fuentes et al. [11] compared the phagocytosis of zymosan particles by RAW 264.7 mouse macrophages in the absence or presence of soluble carbohydrates LAM (from *L. digitata*), mannan (from *S. cerevisiae*), dexamethasone and LPS. The results showed that LAM at a dependent concentration (3.30 to 100 µg/mL) inhibited phagocytosis, the number of particles per cell, and the phagocytic index. In contrast, the presence of another carbohydrate, mannan, had no effect on zymosan phagocytosis by the cells. LAM and dexamethasone suppressed rates of phagocytosis, while inverse results were found with treatment with LPS. These findings confirmed that the LAM is an important ligand identified by macrophages and required for zymosan phagocytosis in naive cells, but not those previously exposed to LPS.

#### 3.1.2. Human Neutrophils

Neutrophils are essential leukocytes of the innate immune system that control infections, especially fungal infections, through their ability to engulf and kill microorganisms [44]. A recent study by our group [5] investigated LAM (from *L. digitata*) and evaluated its immunomodulatory activity within the context of vulvovaginal candidiasis, in relation to the yeast species *Candida albicans* and *C. glabrata*. The results indicated that LAM activated important chemical pathways in neutrophils, such as Reactive Nitrogen Species (RNS) and Reactive Oxygen Species (ROS), including hypochlorous acid, an important microbicidal agent produced by activated leukocytes to control infections, especially fungal infections [45]. The carbohydrate increased the microbicidal activity of these neutrophils, inducing significant oxygen consumption through the NADPH oxidase system, followed by large production of intracellular oxidant species, demonstrated by HClO and dihydrorhodamine-123 assays, as well as the evaluation of the activity of myeloperoxidase, an important enzyme in this process [5].

#### 3.1.3. Other Phagocytic Cells

Sonck et al. [14] investigated the in vitro response of different cells (the monocytes, neutrophils and lymphocytes of pigs), when stimulated by insoluble and soluble glucans, including LAM, from *L. digitata*. Lymphocyte proliferation, ROS production by neutrophils and monocytes, and cytokine production were evaluated. LAM was able to stimulate all the parameters analyzed in the different cells tested. However, stimulation caused by LAM in the different phagocytes was lower than the activity of the other β-glucans. Similar results were reported by Noss et al. [17], who tested 13 different β-glucans, including LAM (from *L. digitata*). LAM was less able to induce the release of interleukins than glucans from other sources. Some authors have suggested that molecular weight is determinant for immunomodulatory activity [46,47,48,49]. However, other factors such as solution conformation and backbone structure, and the degree of branching, are important for immunological effect [14]. Adams et al. [49], suggested that the presence of β-(1,3)-glucose residues in the backbone promote interaction with Dectin-1 molecules and could result in high affinity. Dectin-1 is the main receptor for β-glucans and is present in all potentially phagocytic cells, thus highlighting the β-(1,3)-bond LAM.

### 3.2. Immunomodulation by LAM in Animal Nutrition

Modulation of the immune function by nutrients is an emerging research area in the field of nutrition [50,51,52,53]. Different kinds of LAM vary in their structure and chemical composition, which may modulate the immunological effects on animal performance and gastrointestinal health [14]. In this context, the modulation of immune function by LAM has displayed promising results in the field of nutrition [50]. In vivo experimental models have studied two forms of LAM administration, intravenous and orally. Orally administered LAM can translocate from gastrointestinal (GI) into the systemic circulation, and a peak plasma concentration of approximately three hours was identified with an elimination time similar to intravenous administration [54,55]. Studies report that LAM has been incorporated into the diets of fish, rats and pigs to evaluate immunomodulatory activity.

#### 3.2.1. Fish Model

Studies on stimulation of the immune system in different species of fish have been growing in recent years [56,57,58].

Yin et al. [56] studied the effect of LAM on growth performance, immunological and biochemical parameters, and immune related gene expression in the *Epinephelus coioides* grouper (an important mariculture fish in China and countries of Southeast Asia). The authors found that *E. coioides* fed with LAM had increased immune response gene, cytokine (IL-1b and IL-8) and TLR2 expression. Both pro-inflammatory cytokines and TLRs are linked with the regulation and activation of innate immune response [59,60]. In view of these results, the authors indicate that LAM may also have immunomodulatory activity, increasing non-specific immunity in *E. coioides*.

Guzmán-Villanueva et al. [57] evaluated the dietary effects of LAM from *L. digitata* and Pdp 11 (i.e., *Shewanella putrefaciens*, a probiotic that was isolated from gilthead seabream skin), alone or combined, on growth and humoral factors (i.e., the seric level of total immunoglobulin M (IgM) antibodies, peroxidase and anti-protease activity). These authors also evaluated the cellular innate immune responses (i.e., peroxidase and phagocytic activity of head-kidney leucocytes) and the expression of immune-related genes in gilthead seabream fish (*Sparus aurata*). IgM gene expression presented a tendency towards downregulation in seabream specimens that were fed LAM or LAM + Pdp 11 in their diet, with this significant effect observed after one week of diet exposure. Seric anti-protease activity increased in fish that were fed the combination diet (LAM + Pdp 11). IgM is the primary antibody in fish and a major component of the teleost humoral immune system. IgM is recruited to identify and neutralize foreign antigens, including bacteria and viruses [61]. Moreover, an increase in phagocytic activity was observed after two and four weeks of diet exposure. With regard to cellular immune activity, phagocytosis is a key aspect of innate immunity and part of the first line of cellular defense [61]. When β-glucan receptors are engaged by β-(1,3)- and β-(1,6)-glucans, all immune functions are improved, including phagocytosis, the release of certain cytokines (e.g., IL-1β, TNF-α, IL-6, and interferons), and antigen processing [62]. Additionally, at the gene level, IL-1β and INF-γ transcripts were upregulated in the head-kidney, but only the effects on IL-1β were significant after four weeks in the LAM-fed group. These genes are mainly expressed by natural killer and phagocytic cells, which are in turn the major responders to β-glucans [63]. This study therefore suggests that LAM and Pdp 11 modulate the immune response and stimulate growth of the gilthead seabream.

#### 3.2.2. Rat Model

Neyrinck et al. [64] studied the modulation effect of LAM on the metabolic and toxic response to LPS administration in male Wistar rats, aimed at the alterations of the immune cells present in the hepatic tissue, supporting the importance of nutrients in the control of systemic infection by gram-negative bacteria. To achieve this, the anti-inflammatory prostaglandin PGE_2_ and inflammatory mediators TNF-α and NO_2_ were analyzed. This is important for molecular mechanisms of the progression of sepsis [65]. These authors found that LAM decreased the number of serum monocytes, NO_2_, and TNF-α. They also observed the modulation of intrahepatic immune cells, in which the occurrence of peroxidase-positive cells (monocytes/neutrophils) decreased and the number of ED2-positive cells increased, which corresponded to the resident hepatic macrophages (i.e., Kupffer cells). Thus, the hepatoprotective effect of LAM during endotoxic shock may be linked to its immunomodulatory properties [64].

#### 3.2.3. Pig Model

Smith et al. [15] and Sweeney et al. [16] showed that LAM extracted from different sources was able to reduce colonization by *Enterobacteriaceae* in the ileum and colon in pigs. This was associated with the level of expression of a number of pro-inflammatory cytokine genes in the colon. To evaluate this expression, the ileal and colonic tissues of animals were exposed to LAM through a microbial challenge (absence and presence of LPS). An analysis of cytokine expression revealed significant upregulation of interleukin-6 (IL-6) and IL-8 genes in the colon in LPS-challenged colonic tissue with LAM inclusion [15]. Enhancement of these pro-inflammatory cytokines following LPS challenge is significant for the host, as IL-6 is constitutively expressed by the intestinal epithelium and may play a role in the basal influx of immune cells into the mucosa, epithelial cell growth, homeostasis, and acute inflammation during early immune response. Similarly, IL-8 is responsible for neutrophil chemotaxis and activation at the initial infection site [66,67,68]. Furthermore, Sweeney et al. [16] found that the expression of a number of pro-inflammatory cytokine genes (IL-1α, IL-10, IL-17a and TNF-α) was downregulated in the colon in pigs that were exposed to LAM from *L. digitata* and *L. hyperborea*. While dietary exposure to Laminarin did not stimulate pro-inflammatory cytokine production in the gastric mucosa, it enhanced the LPS-induced production in colonic tissue. One possibility is that LAM molecules coordinate signaling between Toll-like receptors (TLRs) and non-TLRs during immune stimulation [69]. LAM binds to mammalian non-TLRs receptors (dectin-1, complement receptor 3, lactosylceramide, scavenger receptors) stimulating innate immunity through the activation and proliferation of defense cells [70], consequently increasing cytokine production, phagocytosis, oxidative bursts, activation of the alternative complement pathway and the release of lysosomal enzymes [71]. The authors suggested that the inclusion of LAM from *L. digitata* in the diet may enhance pro-inflammatory response to a microbial challenge, since a general microbial challenge can be extrapolated from an LPS challenge.

Ryan et al. [72] concluded that dietary supplementation with LAM from *L. hyperborea* and *L. digitata* and β-glucan from *S. cerevisiae* significantly decreased the gene expression of Th17-related cytokines (IL-17a, IL-17F, and IL-22), the IL-23 receptor, and IL-6 in the porcine colon. No alterations in the regulatory T (TREG) cell-related target Foxp3 or transforming growth factor β (TGF-β) were observed, although a significant reduction of IL-10 was found in the *L. digitata*-supplemented group. Data suggested that Th17 cells have an important role in host defense against specific pathogens and are potent inducers of autoimmunity and tissue inflammation [73]. The upregulation of the Th17 inflammatory response has been highlighted as a major contributor to the underlying pathology of inflammatory bowel disease, whereas TREG cells have been highlighted as pivotal in suppressing autoimmune and inflammatory responses in the gut [72,73].

Other possibilities of study of LAM in the diet of pigs include the combined use of extracts from different species of *Laminarin* spp. and also the combination of LAM with other compounds. LAM and FUC are the main water-soluble polysaccharides of brown algae and extracts containing the two have been explored as a novel source of bioactive compounds containing immunomodulatory and antimicrobial properties [24].

Reilly et al. [25] investigated the effects of LAM and FUC from *L. hyperborea* and *L. digitata* seaweed extract on immune status of weaned pig, as well as on other factors. Supplementation with the two types of LAM alone or in combination had no significant effect on the expression of the panel of cytokines evaluated, with only an increase in interleukin 8 (IL-8) mRNA detected. The authors indicate that the reason for this increase in IL-8 with the combination of seaweed extracts is unclear as the individual seaweed extracts did not increase IL-8 gene expression. One hypothesis is that the combination of two types of LAM may act synergistically to induce IL-8 expression due to the combination of insoluble (from *L. hyperborea*) and soluble LAM (from *L. digitata*).

The factorial arrangement of isolated LAM and FUC and combinations of the same at different concentrations (0–300 parts per million (ppm) or mg/kg LAM and 0–240 ppm or mg/kg FUC) downregulated the expression of pro- and anti-inflammatory cytokines (IL-6, IL-17a, IL-1β, and IL-10) in the colon of pigs offered only LAM-supplemented diets. However, this effect was lost when combined LAM and FUC were used. The authors associated the effect of LAM to a modulation of the intestinal microbiota, in addition to resulting in an improvement in growth performance [26,27].

Leonard et al. [74] showed the effects of maternal dietary supplementation with seaweed extract (SWE) containing LAM and fucoidan and fish oil (FO) from Day 109 of gestation until weaning of the piglets (Day 26). Their results demonstrated that SWE dietary supplementation is responsible for the increase of IgG concentration in colostrum, and serum on Days 5 and 12 in piglets suckling SWE-supplemented sows. This is beneficial to the host defense against invading pathogens and may be related to LAM due to the activation of dectin-1 receptors normally expressed on the cell surface of monocytes, macrophages, and neutrophils, stimulating host immune function [3,75]. In 2011 [76], studying the effect of the same dietary supplementation from Day 109 of gestation until weaning (Day 26) on post-weaning (PW) pig performance, the authors demonstrated that SWE induced a significant increase of TNF- α in ileal and TFF3 mRNA in the colon of pigs nine days PW. TFF3 can stimulate the migration of epithelial cells and the maintenance of the surface of the intestinal mucosa barrier [77]. Therefore, the increase of TFF3 may benefit the restoration of the mucosa, stimulating the migration of epithelial cells, an important factor in this study due to the transient gut inflammation identified. In addition, Leonard et al. [78] investigated the effect of maternal dietary supplementation with SWE from Day 107 of gestation until weaning (Day 26), showing enhanced IgA and tended to increase IgG concentrations, in colostrum. The authors evaluated also at weaning after at an ex vivo *Escherichia coli* LPS tissue challenge. An increased of ileum TNF-α mRNA expression in piglets suckling SWE-supplemented sows was observed. Primarily, through the recruitment of eosinophils, neutrophils, and macrophages to the site of infection, TNF-α is directly linked to the cellular immunity of the host defense [79].

### 3.3. Immunomodulation by LAM in Microorganism-Host Interaction

Infection pathogenesis is a result of a continuous interaction between host and microbial pathogens, and the balance of this relationship keeps the microorganism in a state of colonization or infection. New insights into the characterization of the host immune response to pathogens emerge annually, connected to the development of new strategies to modulate the immune system against infecting agents. In this context, LAM contributes satisfactorily, since several in vitro and in vivo studies have shown that depending on the biochemical characteristics of the LAM and the concentration used, this carbohydrate modulates the immune response when professional phagocytes are challenged.

Cheng et al. [6] investigated the antioxidant activity of LAM in a model of sepsis in rats (Sprague Dawley) and evaluated its effect on oxidative stress in the lungs and lipid peroxidation. Sepsis is an example of imbalance between the molecular mechanisms of ROS production and cellular antioxidants [65]. The concentration of LAM used (200 or 400 mg/kg body weight, administered by gavage) modulated the result of the interaction of microorganisms in the sepsis model. LAM polysaccharides significantly normalized catalase activity, increased glutathione peroxidase and superoxide dismutase activity, and decreased malondialdehyde concentration (the end product of lipid peroxidation). According to these authors, LAM polysaccharides appeared to more effectively reduce sepsis-induced oxidative stress and lipid peroxidation in rats.

Kuda et al. [37] investigated the effects of products of LAM (SPS, EE fractions of frond (kombu) and holdfast (ganiashi)) on *Listeria monocytogenes* in two cellular models, human enterocyte-like Caco-2 cells and murine macrophage RAW 264.7 cells. The microorganism studied is important because it can bring risks to human health, mainly food poisoning related to the consumption of ready-to-eat foods. The results suggest that LAM intake, particularly with respect to ganiashi, can prevent *L. monocytogenes* enterogastric invasion and infection due to its relationship with NO production. It is well established that NO is a highly reactive free radical responsible for numerous organ specific regulatory functions, as well as a cytotoxic agent in the immunological interaction between invading microorganisms and macrophages [80,81].

Recently, Bouwhuis et al. [82] highlighted the problem of the commercial pig trade and the early weaning of the animals, which triggers a serious imbalance of intestinal homeostasis, among other problems. ZnO is currently recommended to reduce the severity of diarrhea and improve growth performance post-weaning. However, there exist concerns in relation to the high inclusion rates and Zn accumulation in the soil. This explains the strategy of using LAM and ZnM (zinc methionine), together or otherwise, for intestinal microbiota homeostasis. Orally introduced LAM together with ZnM reduced the abundance of attaching and effacing *Escherichia coli* (AEEC), but dietary treatments had no effect on the abundance of *Enterobacteriaceae* or *Lactobacillus* spp. in the caecum, colon or rectum (*p* > 0.10). Diet supplemented with LAM increased y-interferon (IFN-γ) in the ileal tissue. β-glucans can bind (receptors) and stimulate activity to a variety of immune cells (macrophages, natural killer cells and neutrophils) initiating the development of the adaptive immune system and stimulating T-cell-specific responses through the induction of cytokines like IFN-γ. More recently [83], the same authors and collaborators investigated the effects of the dietary supplementation of galactooligosaccharides (GOS) and seaweed extract containing LAM and FUC (SWE) on intestinal health parameters after in vivo *Salmonella typhimurium* challenge. Dietary suplementation with SWE resulted in reduced fecal shedding of *Salmonella typhimurium* at day seven post-challenge, while GOS supplementation increased colonization by *Lactobacillus* spp. in colonic and caecal digesta samples. Meanwhile, both dietary supplements down-regulated pro-inflammatory cytokine expression (IL-6, IL-22, TNF-α and Reg3-γ). Thus, dietary supplementation with SWE or GOS, were able to balance the bacterial flora and to modulate the intestinal immune system.

Vulvovaginal candidiasis (VVC) is also defined by the imbalance in the host–yeast interaction. In this context, our research group sought to know more about the innate immune response in VVC of women with different symptoms. Clinical isolates of *Candida albicans* [84] and *C. glabrata* [5] from women with recurrent VVC (RVVC) exhibited different behavior in the presence of human neutrophils. *C. albicans* were resistant to the action of neutrophils, mainly by releasing detoxification enzymes, while *C. glabrata* were susceptible to the killing action. Neutrophils treated with LAM were able to increase oxidant species production (as previously explained), and modulate the release of cytokines by decreasing some pro-inflammatory cytokines such as IL8, IL1-β and tumor necrosis factor (TNF-α). These results were independent of the challenge of different yeast species, showing that LAM was able to modulate the immune response of neutrophils to act in sensitive and even potentially resistant clinical isolates [5].

## 4. Antitumor Activity of LAM

Another feature of LAM is antitumor activity and relatively few sideeffects (Table 2).

Park et al. [85] examined the mechanisms through which LAM (from *L. digitata*) effects HT-29 cells and analyzed its effect on the insulin-like growth factor (IGF-IR) signaling pathway. Subsequently [18], the same research group analyzed the mechanisms through which LAM (from *L. digitata*) induces apoptosis in HT-29 colon cancer cells and the involvement of the ErbB signaling pathway. In summary, cell viability assay showed that LAM dose-dependently induced cell death, causing apoptosis in highly proliferative cancer cells. Cell death seems to be induced by decreased mitogen-activated protein kinases (MAPK) and ERK phosphorylation expression, associated with IGF-IR [86]. In addition, it was found that LAM is also capable of modulating the proliferation and survival of colon cancer cells by regulating the ErbB receptor signaling pathway. The *Erb2* gene is an important regulator of aberrant growth in colon cancer. In this way, the authors suggested the potential of LAM as a bio-functional food with anticancer effects on human colon cancer.

Recently, Song et al. [87] studied the effects of LAM (derived from *L. digitata*) on the maturation of dendritic cells and on the in vivo activation of anticancer immunity. The LAM-induced maturation of spleen and tumor draining lymph nodes (drLN) and dendritic cells (DCs) in in vivo tumor microenvironments were investigated, and the results showed that the laminarin-induced maturation of both CD8α+ and CD8α cDCs promoted antigen (Ag) specific T helper 1 (Th1) and cytotoxic T lymphocyte (CTL) immune responses. The combination of LAM and ovalbumin (OVA) inhibited the metastasis of B16-OVA melanoma cells in mice livers in vivo by activating OVA specific IFN-γ production. Moreover, the combination of LAM and OVA induced specific killing of OVA-pulsed splenocytes in tumor-bearing mice, which indicates that this combination promoted OVA specific CTL activation. The authors suggested that LAM is a novel immune-stimulating reagent that can induce DC maturation and Ag specific Th1 and CTL activation that can effectively kill Ag-expressing B16 melanoma cells in vivo.

Other authors have reported the potential effects of purified LAM polysaccharides on various kinds of cancer. Zhai et al. [88] investigated the antitumor effects of a sulfated polysaccharide fraction of *L. japonica* (LJSP) on cervical carcinoma. They found that LJSP exerted inhibitory effects on five tumor cell lines: HeLa (cervical carcinoma), U14 (cervical carcinoma), A549 (lung carcinoma), Bel-7402 (hepatoma), and HCT-8 (colon carcinoma). The cervical carcinoma cell lines were more sensitive to LJSP exposure than the other cell lines. However, in vivo tests showed that LJSP significantly inhibited the growth of the U14-implanted tumor and markedly induced the apoptosis of tumor tissue cells by modulating the Bax/Bcl-2 ratio, which plays a crucial role in apoptosis [89].

Other polysaccharides that were purified from aqueous extracts of *L. japonica* (denominated novel polysaccharide WPS-2-1) were evaluated by Peng et al. [19]. A cytotoxicity assay showed that WPS-2-1 presented significant antitumor activity against BGC823 and A375 carcinoma cells in vitro. No anti-proliferative effects of WPS-2-1 on aortic vascular smooth muscle cells were observed, implying that these polysaccharides had no direct cytotoxic effects on non-cancer cells. These authors [90] also investigated the mechanism of antitumor effects of *L. japonica* polysaccharide WPS-2-1 on A375 cells in vitro. They found that WPS-2-1 induced apoptosis in A375 cells. WPS-2-1-induced apoptosis was associated with alterations in the expression of Bcl-2 family proteins. The mitochondrial apoptotic pathway appeared to be involved in WPS-2-1-induced apoptosis, which included the loss of the mitochondrial membrane and the activation of caspase-3/9. Bcl-2 family proteins play central roles in regulating cellular apoptosis [91].

Kim et al. [92] described the use of laminarin polysaccharide 1 (LP1) from *L. japonica* and LP1-derived oligosaccharides (LOs) to inhibit cellular apoptosis in mouse thymocytes. Thymocytes treated with LO and LP1 remained alive in culture for up two weeks, while the control group (culture unsupplemented medium) survival was only three days. Thymocyte survival was dose-dependent, showing that the interaction of LO and LP1 with these cells can suppress apoptotic cell death.

## 5. LAM as Vaccine

As described above, β-glucan itself can exert broad anti-infective effects. Furthermore, LAM has also been shown to have protective effects against different microorganisms, such as bacterial infections [93], oral microbial species [94], *Listeria monocytogenes* [38], and *Candida albicans* [5,10]. β-glucan was also found to be recognized by neutrophil and polymorphonuclear leukocytes in response to *C. albicans* infection [95]. The basic molecular structures of β-glucan polymers are similar and highly conserved among different pathogenic fungal species, mainly consisting of β-(1,3)- and β-(1,6)-linked repeating units of d-glucose cross-linked together, and complexed variously with chitin and other glycoproteins [96]. This mechanism that allows the recognition and responses to conserved structural components, particularly β-glucans, has evolved in mammals as a defense against fungal pathogens [10]. Research has shown that LAM with β-glucan from a non-fungal source may contribute to possible vaccination against different fungi (Table 3), while more recently the use of proteins, derived from different bacteria, were conjugated to the polysaccharide LAM model and tested in mice for their ability to induce antibodies against the carbohydrate antigen [93,97].

Numerous β-glucan protein conjugate vaccines have presented efficacy in experimental models of candidiasis, aspergillosis, and cryptococcosis [98]. Studies of LAM from the brown alga *L. digitata*, conjugated with the genetically inactivated diphtheria toxin CRM197, have found that it is an interesting alternative for inducing the production of anti-β-glucan antibodies, which are capable of conferring protection against all three of the above infections [21,22,99].

Torosantucci et al. [21] used LAM conjugated to the diphtheria toxoid CRM197 (i.e., a carrier protein that is used for some glycoconjugated bacterial vaccines). Bromuro et al. [20] formulated LAM-CRM197 conjugated to MF59 (i.e., a human-acceptable adjuvant). This LAM-CRM197/MF59 conjugate proved to be immunogenic and protective as an immunoprophylactic vaccine against both systemic and mucosal infections that were caused by *C. albicans*. Furthermore, LAM-CRM 197-vaccinated mice were protected from a lethal challenge of *Aspergillus fumigatus* conidia, demonstrating that this novel conjugate vaccine efficiently immunized the mice and protected them from two major fungal pathogens through mechanisms that may include the direct antifungal properties of anti-β-glucan antibodies [20,21,22].

However, the vaccine design that was used in these previous investigations had some limitations, such as the relative complexity of the β-glucan antigen that was used in the conjugate. It was difficult to discern from the LAM-CRM197 vaccine which were the most protective β-glucan epitopes. One consideration is that only some anti-β-glucan antibodies may have protective value. A monoclonal antibody that exclusively recognized the β-(1,3)-glucan epitope and not both β-(1,3)- and β-(1,6)-glucans exhibited protective effects in passive vaccination studies [20,21,22].

One issue, therefore, is whether the presence or arrangement of this β-(1,6) branching on the LAM molecule impacts the immune response that is induced by the glycoconjugate in terms of antibody specificity and/or protective effects. Bromuro et al. [20] studied a series of different β-glucan-based conjugated vaccines, replacing LAM with β-glucan molecules with the absence of, or defined number and positions of, β-(1,6) branching, using: (i) a natural β-glucan curdlan (Curd) from *Alcaligenes faecalis* (Curd-CRM197); (ii) conjugates of a synthetic β-glucan linear with 15 repeats (15mer-CRM197); and (iii) synthetic conjugates with β-(1,6)- and β-(1,3)-branched β-glucan oligosaccharides with a branching point every five repeats (17mer-CRM197). It was found that the protective β-glucan epitope was conformationally defined, and this protective conformation was seen with LAM-CRM197, Curd-CRM197, and 15mer-CRM197, but not with 17mer-CRM197, indicating the possibility of developing a synthetic vaccine against fungal diseases, thus replacing the highly heterogeneous LAM polysaccharide with a synthetic molecule. Despite this progress with β-glucan as a potential vaccine against human pathogenic fungi, further studies are needed to define the structural requirements of β-(1,3)-glucan oligosaccharide–protein conjugates as candidate vaccines.

## 6. New Perspectives of the Application of LAM

Among the numerous possible applications of LAM for improving host (animal or human) response, fungal infections offer an excellent alternative, as most are results of the imbalance of fungi–host interaction.

Vulvovaginal candidiasis (VVC) is the first or second most frequent female genital disorder, affecting millions of women every year around the world, and has been considered an important public health problem. VVC is an old disease which remains a challenge in the modern world as, despite therapeutic advances, treatments are not always effective [100]. Yeasts from the *Candida* genus, especially *C. albicans*, can colonize the vaginal mucosa without any symptoms, but an increased rate of vaginal colonization often evolves into the development of symptoms, resulting in VVC or RVVC, defined by as at least three or four symptomatic episodes in a 12 months period [101]. The evolution from *Candida* vaginal colonization to VVC is usually attributed to the disruption of the balance between the yeast and host environment due to physiological or non-physiological changes [102]. Meanwhile, several host-related and behavioral risk factors for RVVC have been identified [103]. Information about the current state of the art on VVC, and the risk factors, epidemiology and microbiology of VVC, as well as *Candida* virulence factors, is today well known. Nevertheless, knowledge regarding the mechanism associated with vaginal pathogenicity and the overall reason why yeasts evolve from colonization to pathogenicity still requires improvement. In addition, the area of gynecology needs to advance in terms of search options to recover the efficiency of human cells to combat these pathogens. This imbalance must therefore provide clues to allow the creation of new treatment approaches using LAM. We have previously shown that LAM exerts a positive in vitro modulation on the phagocytic activity of human neutrophils on *C. albicans* isolated from RVVC. It is known that antifungal therapy is highly effective for individual symptomatic attacks of VVC but does not prevent recurrences (RVVC) [104]. Thus, we strongly believe that β-glucan, especially LAM, can act as a therapeutic modulator for VVC, through direct therapeutic options such as active or passive vaccination or indirect options such as immunoregulation. In this way, it seems possible that women could be protected by the local or even systemic infusion of LAM and the subsequent increase of innate and adaptive host immunity. We also believe that modulation may go beyond innate immune response stimulation, and can also increase the tolerance of the first encounter of the epithelial cells with the yeast. Furthermore, considering the increasing rate of antifungal resistance, β-glucan might be used in combination with classical fungicidal drugs to induce a better host response.

Another interesting model for future perspectives of application of LAM is paracoccidioidomycosis (PCM), which is the most important systemic mycosis in Latin America, with Brazil having the most endemic areas for this disease in the world. PCM is considered a major health problem in Brazil, as it is the eighth most common cause of death among chronic/recurrent infectious and parasitic diseases [105].

In humans, PCM infection occurs after contact with the *Paracoccidioides* spp. fungus. However, the evolutionary process is multifactorial and involves fungal virulence potential and host immune response [106]. Individual differences in the host pattern [107] of immune response to the fungus determine the evolution of the disease, which has a wide spectrum of clinical manifestations, ranging from benign localized to severe disseminated forms, with high mortality rates. According to the immunological status of the patient, infection without disease, localized disease, or systemic disease may occur. The patient outcome depends on the genetic patterns and immunity, with a balance of CD4/CD8 regulating the secretion of cytokines of the Th1 and Th2 type, which correlates with the resistance or susceptibility of the host [108].

The use of animals in research is essential for studies of host–fungal interaction. Many species of vertebrate and invertebrate animals have been evaluated and the murine model is considered the gold standard for in vivo studies simulating *Paracoccidioides* spp. infection [108]. It can mimic the benign and severe forms of the human disease, and has been used to supply studies on the immunopathology of human PCM. Susceptible mice present inefficient macrophage activation and a progressive form of the disease. In contrast, resistant mice present efficient macrophage activation, resulting in the resolution of the infectious process.

Considering the increasing number of virulence mechanisms that have been identified, especially in *P. brasiliensis*, the ability of this fungus to induce apoptosis in pulmonary epithelial cells, during the early phase of experimental murine paracoccidioidomycosis [109,110], is highly relevant. This competency could be considered as a phenotypic marker of resistance against host defense mechanisms [111]. The cellular immune response in PCM encourages research into treatment with β-glucans, such as LAM. Pulmonary macrophages and dendritic cells are the first cells to interact with the fungus and biochemical mediators such as nitric oxide, which are produced mainly by INF-γ-activated macrophages and are fungicidal to *Paracoccidioides* spp. [112]. In this review, we have shown several studies highlighting the ability of LAM to modulate the activation of phagocytes, and increasing the production of oxygen and nitrogen reactive species in the macrophages [5,15,37]. It is known that *Paracoccidioides* spp. can cope with oxidative and nitrosative stress, because the enzymes present such as catalases, peroxidases, thioredoxin and superoxide dismutases can detoxify the immune response of the macrophages [112]. However, our studies [10,84] found that LAM can stimulate professional phagocytes to produce larger amounts of chemical mediators and overcome the detoxification capacity of some fungal strains. In addition, the activation of Dectin-1 by LAM may stimulate other biochemical processes such as the increased recruitment of leukocytes and phagosome maturation leading to more rapid phagocytosis [113]. Thus, intervention with LAM in this pathway may prevent *Paracoccidioides* spp. escaping from the immune system, or prevent cases of chronicity and the formation of classical pulmonary granulomas.The effect of LAM on human defense cells against experimental *Paracoccidioides* spp. infection or in vitro assays therefore deserves to be investigated.

## 7. Conclusions

In summary, in vitro and in vivo studies have shown that LAM from seaweed of the genus *Laminaria* and its derivatives can induce an initial oxidative burst and the activation of numerous interleukins, resulting in the modulation of the immune system, triggering benefits for health and also the increase of the microbicidal potential of phagocytes. LAM can also activate signaling pathways, pre-activate dormant immune cells, and act as a priming agent. Discrepancies in literature regarding the effects of LAM may be attributable to differences in experimental conditions, including animal models, cell types, and phagocytic targets. We understand that LAM is a model of great relevance and deserves to be valued in studies of host interaction in several biological models. However, we note that some studies did not specifically state from which species the LAM was derived, making further classification of the biological effects difficult.

Another feature of LAM is related to the induction of cancer cell death. Recent research in this area has been promising, showing that LAM acts in a dose-dependent manner and causes apoptosis of these cells with no direct cytotoxicity in non-cancer cells.

Research into LAM containing branched polysaccharides including both β-(1,3)- and β-(1,6)-linked d-glucose sequences and β-glucan from non-fungal sources has demonstrated that LAM conjugates with adjuvants may contribute to possible vaccines that can induce the production of anti-β-glucan antibodies, which are capable of conferring protection against different pathogenic fungi. Recent studies suggest replacing LAM with a synthetic β-glucan molecule with a less heterogeneous LAM polysaccharide. Nonetheless, further studies with β-glucans (LAM or a synthetic β-glucan molecule) are needed to evaluate their potential as vaccines against human pathogenic fungi.

Finally, the biological effects of LAM on different signaling pathways indicate that this carbohydrate goes beyond its classic effects as solely an immunomodulator with encouraging therapeutic prospects.

## Figures and Tables

**Table 1:** Immunomodulatory properties of β-glucans from genus *Laminaria* spp.

Source	Product	Immunomodulation	Type of Study	Reference
*Laminaria digitata*	Laminarin	- Increase in the release of H_2_O_2_, calcium, NO, MCP-1, VEGF, leukemia inhibitory factor, and G-CSF - Enhancement of expression of STAT1, STAT3, Jun, Fos, and COX-2 mRNA	In vitro (murine RAW 264.7 macrophages)	[38]
*Laminaria japonica*	Kombu and ganiashi	- Ganiashi SPS increased NO production	In vitro (Caco-2 cells and RAW 264.7 macrophages)	[37]
*Laminarina digitata*	Laminarin	- Increase in oxidant and nitrogen species production - Reduction of pro-inflammatory cytokines IL8, IL1-β and TNF-α	In vitro (human neutrophils and total leukocytes)	[5]
		- Inhibition of phagocytosis	In vitro (murine RAW 264.7 macrophages)	[11]
		- Weak ROS production - Weak lymphocyte proliferation stimulus	In vitro (monocytes, neutrophils, and lymphocytes in pigs)	[14]
		- Weak production of cytokines IL-1β, IL-6, IL-8, and TNF-α	In vitro (whole blood cultures)	[17]
-	Laminarin	- Enhancement of expression of the genes cytokines (*IL-1b*, *IL-8*) and *TLR2*	In vivo (fish, *Epinephelus coioides*)	[56]
*Laminarina digitata*	Laminarin	- Increased phagocytic activity - Enhancement of expression IL-1β and IFN-γ transcripts - Increased serum IgM level	In vivo (fish, *Sparus aurata*)	[57]
Brown Algae	Laminarin	- Decrease in number of serum monocytes, NO_2_, and TNF-α, after challengewith LPS - Modulation of intra-hepatic immune cells	In vivo (Wistar rats)	[64]
-	Laminarin polysaccharides	- Increased GPx and SOD activity, after sepsis - Reduction of MDA concentrations, after sepis	In vivo (Sprague-Dawley rats)	[6]
*Laminaria digitata*	Laminarin	- Enhancement of IL-6 and IL-8 cytokine gene expression	In vivo (pig)	[15]
*Laminaria digitata* and *L. hyperborea*	Laminarin	- No stimulation of any pro- or anti-inflammatory cytokines in ileum. - Down-regulation of anti-inflammatory cytokines gene expression (*IL-1a*, *IL-10*, *TNF-α*, and *IL-17a*) in the colon. - Reduction of pro-inflammatory markers in the colon	In vivo (pig)	[16]
		- Decreased *IL-17a*, *IL-17F*, and *IL-22* gene expression in inflammatory bowel disease	In vivo(pig)	[72]
		- Increase in *IL-8* gene expression, *L. digitata* + *L. hyperborea*	In vivo (pig)	[25]
*Laminaria* spp.	SWE (Laminarin + Fucoidan)	- Increased IgG concentrations, in colostrum	In vivo (pig)	[74]
		- Increase *TNF-α* gene expression, in the ileum - Increase *TFF3* gene expression, in the colon	In vivo (pig)	[76]
		- Increase *TNF-α* gene expression, after LPS challenge	In vivo (pig)	[78]
	Laminarin + Fucoidan	- Lower expression of IL-6, IL-17a, and IL-1-β mRNA, in the colon, when using only LAM	In vivo (pigs)	[26,27]
	Laminarin	- Increased IFN-γ, in the ileum	In vivo (pigs)	[82]
	Laminarin + Fucoidan	- Down-regulated IL-6, IL-22, TNF- α and *Reg3-γ* gene expression	In vivo (pigs)	[83]

**Table 2:** Antitumor activity of β-glucans from seaweed of the genus *Laminaria* spp.

Source	Extract	Applicability	Type of Study	Reference
*Laminaria digitata*	Laminarin	- Dose-dependent induction of cell death - Increase in the percentage of cells in the sub-G1 and G2-M phases - Inhibition of heregulin-stimulated phosphorylation of ErbB2 - Decrease in cellular proliferation	- In vitro (HT-29 colon cancer cells)	[18]
*Laminaria japonica*	Sulfated polysaccharide fraction (LJSP)	- Highest inhibitory effect on cervical carcinoma U14 cells among five tumor cell lines - In vivo, LJSP inhibited tumor growth and enhanced spleen and thymus indices and bodyweight of U14 tumor-bearing mice - Prominent antitumor activity and low toxic effects	- In vitro (HeLa (cervical carcinoma), U14 (cervical carcinoma), A549 (lung carcinoma), Bel-7402 (hepatoma), and HCT-8 (colon carcinoma) cells) - In vivo (female Kunming mice, 6–8 weeks old; weight, 18–22 g)	[88]
	Novel polysaccharide WPS-2-1	- Dose-dependent antitumor activity against A375 and BGC823 cells - Lower cytotoxic effects on vascular smooth muscle cells	- In vitro (human gastric carcinoma cell line BGC823, human melanoma cell line A375, and aortic vascular smooth muscle cells)	[19]
	Novel polysaccharide WPS-2-1	- Induction of apoptosis associated with alterations in the expression of Bcl-2 family proteins - Mitochondrial apoptotic pathway involvement in WPS-2-1-induced apoptosis, which included the loss of the mitochondrial membrane and activation of caspase-3/9 - Effective inhibition of proliferation of A375 cells in vitro and induction of apoptosis via mitochondrial apoptotic pathway	- In vitro (human melanoma cell line A375)	[90]
	Laminarin polysaccharides (LP1)	- Suppression of apoptotic death around three- or two-fold - Prolonged cell survival in culture at a rate of 20–30%	- In vitro (mouse thymocytes)	[92]

**Table 3:** Applicability of β-glucans from seaweed of the genus *Laminaria* spp. in vaccines against different pathogenic fungi.

Source	Extract	Applicability	Type of study	Reference
*Laminaria digitata*	Laminarin	Vaccine against *C. albicans* and *A. fumigatus*	- In vivo (female CD2F1 mice, 4 weeks old; Harlan)	[21,22]
		Vaccine against *C. albicans*	- In vivo (female CD2F1 mice, 4 weeks old; Harlan-Nossan)	[20]
		Vaccine against *C. albicans*	- In vivo (female BALB/c and BALB/cnu/nude mice, 6–8 weeks old)	[97]
		- Increase in levels of TGF-β and IL-6 - High titers of Ab that recognizes *C. albicans* β-mannan Ag	- In vitro (mouse RAW264.7 macrophages; bone marrow-derived DCs [BMDCs])	[12]

## Abbreviations

LAM	Laminarin or Laminaram
SPSs	Soluble polysaccharides
EE	Ethanolic extract
LPS	Lipopolysaccharide
H_2_O_2_	Hydrogen peroxide
NO	Nitric oxide
MCP-1	Monocyte chemotactic protein-1
VEGF	Vascular endothelial growth factor
G-CSF	Granulocyte-colony stimulating factor
MIP-1α	Macrophage inflammatory proteins
COX-2	Cyclooxygenase-2
RNS	Reactive Nitrogen Species
ROS	Reactive Oxygen Species
HClO	Hypochlorous acid
IgM	Immunoglobulin M
IGF-IR	Insulin-like growth factor
MAPK	Protein kinases
drLN	Draining lymph nodes
DCs	Dendritic cells
Ag	Antigen
Ab	Antibodies
OVA	Ovalbumin
Th1	T helper 1
